# Anti-tumour activity of ICI 118630, a new potent luteinizing hormone-releasing hormone agonist.

**DOI:** 10.1038/bjc.1979.50

**Published:** 1979-03

**Authors:** R. I. Nicholson, P. V. Maynard

## Abstract

Experiments were undertaken with DMBA-induced mammary tumours of the rat to determine the anti-tumour properties of a new and potent luteinizing hormone releasing hormone (LH-RH) agonist, [D-Ser(But) 6Azgly10]-LH-RH (ICI 118630). Tumours were classified according to their oestrogen-receptor (ER) content. Twice daily i.m. injections of either 5 micrograms or 0.5 micrograms ICI 118630 in saline were as effective as ovariectomy or tamoxifen therapy in causing the regression of ER+ DMBA-induced mammary tumours. ER- mammary tumours showed a more equivocal overall response to ICI 118630, some tumours progressing, others regressing. About one-third of the ER+ tumours disappeared in the 20-day treatment period. Those tumours which did regrow after the cessation of treatment proved to be hormone-dependent. In addition to the inhibitory effects of the LH-RH agonist on pre-existing tumours, ICI 118630 also reduced the total number of new tumours formed during and after treatment.


*Br. J. Cancer* (1979) **39**, 268

### ANTI-TUMOUR ACTIVITY OF ICI 118630, A NEW POTENT LUTEINIZING HORMONE-RELEASING HORMONE AGONIST

R. I. NICHOLSON AND P. V. MAYNARD

*From the Tenovus Institute for Cancer Research, Welsh National School of Medicine, Heath Park, Cardiff*

Received 9 October 1978 Accepted 17 November 1978

**Summary.—Experiments were undertaken with DMBA-induced mammary tumours of the rat to determine the anti-tumour properties of a new and potent luteinizing hormone-releasing hormone (LH-RH) agonist, [D-Ser(Bu$^t$) $^6$Azgly$^{10}$]-LH-RH (ICI 118630). Tumours were classified according to their oestrogen-receptor (ER) content. Twice daily i.m. injections of either 5 $\mu$g or 0·5 $\mu$g ICI 118630 in saline were as effective as ovariectomy or tamoxifen therapy in causing the regression of ER$^+$ DMBA-induced mammary tumours. ER$^-$ mammary tumours showed a more equivocal overall response to ICI 118630, some tumours progressing, others regressing. About one-third of the ER$^+$ tumours disappeared in the 20-day treatment period. Those tumours which did regrow after the cessation of treatment proved to be hormone-dependent. In addition to the inhibitory effects of the LH-RH agonist on pre-existing tumours, ICI 118630 also reduced the total number of new tumours formed during and after treatment.**

ONE OF THE MOST IMPORTANT characteristics of mammary tumours induced in rats by dimethylbenz(a)anthracene (DMBA) is that most of them are hormone-dependent and regress after removal of the ovaries (Huggins *et al.*, 1959), the pituitary gland (Daniel & Pritchard, 1963) or in response to anti-oestrogens (Nicholson & Golder, 1975). The tumour, therefore, represents a good model with which drugs previously shown to interfere with either the production or secretion of ovarian or pituitary hormones can be screened for anti-tumour activity.

The present report investigates the anti-tumour properties of one such compound, [D-Ser(Bu$^t$)$^6$Azgly$^{10}$]-luteinizing hormone-releasing hormone (ICI 118630), a new and potent luteinizing hormone-releasing hormone (LH-RH) agonist (Dutta *et al.*, 1978*a*; Nicholson *et al.*, 1978). Administration of ICI 118630 at high dose levels (0·5 and 5 $\mu$g twice daily) in rats reduces plasma oestradiol levels (Maynard & Nicholson, 1979) and decreases the weight of the uterus in both human-chorionic-gonadotrophin-treated (Dutta *et al.*, 1978*a*) and untreated animals (Maynard & Nicholson, 1979).

### MATERIALS AND METHODS

*Peptide.*—ICI 118630 was synthesized by solution methods (Dutta *et al.*, 1978*b*) by Dr A. S. Dutta, ICI Pharmaceuticals Division, Macclesfield, England. The purity of the sample was >95%, as assessed by paper electrophoresis, thin-layer chromatography and amino-acid analysis.

*Animals.*—Mammary tumours were induced in virgin female Sprague–Dawley rats (55±2 days old) by intubation with a single dose of 20 mg DMBA in 1 ml sesame oil. Animals were housed in groups of 5 in a 12 h light/12 h dark environment and fed diet and water *ad libitum.* After 5 weeks, the rats were palpated for tumours at weekly intervals and the size recorded as the mean of 2 perpendicular diameters, one measured across the greatest width. Tumour volume was estimated using the formula $\frac{4}{3}\pi r^3$ where $r$ is the mean radius.

*Treatments.*—When tumours reached an approximate volume of 1·76 cm$^3$ (1·5 cm mean diameter) a small portion (100 mg) of each tumour was removed under ether anaesthesia and stored at −20°C for oestrogen receptor (ER) assay. The remainder of the tumour was left *in situ* and the animals divided into 6 groups which were treated for 20 days as follows:

Group (a), 11 animals bearing 18 tumours received twice daily i.m. injections of 5 μg ICI 118630 in saline (100 μl);
(b), 7 animals bearing 9 tumours received twice daily injections of 0·5 μg ICI 118630;
(c), 8 animals bearing 12 tumours received twice daily injections of 0·05 μg ICI 118630;
(d), 7 animals bearing 13 tumours received twice daily injections of saline;
(e), 9 animals bearing 12 tumours were ovariectomized at Time 0 and given twice daily injections of saline;
(f), 9 animals bearing 12 tumours received daily i.m. injections of tamoxifen [*trans* isomer of 1-(p-β-dimethylaminoethoxyphenyl)-1,2-diphenylbut-l-ene] (100 μg) in sesame oil (100 μl).

After the injections had been completed, regrowth of tumours was monitored at weekly intervals. Animals bearing reactivated tumours from Groups (a–c) and (f) were ovariectomized when at least one tumour per animal reached a tumour volume >8·2 $cm^3$ (2·5 cm mean diameter).

The procedures for the estimation of the

FIG. 1.—Response of oestrogen-receptor-positive ($ER^+$) mammary tumours to treatment with ICI 118630 and tamoxifen. 5 groups of animals were injected twice daily for 20 days with either (a) 5 μg ICI 118630, (b) 0·5 μg ICI 118630, (c) 0·05 μg ICI 118630, (d) saline or (e) saline after ovariectomy. The 6th group (f) received daily i.m. injections of tamoxifen (100 μg/injection). Tumour growth patterns were recorded as changes in tumour volume.

ER content of tumour biopsy specimens, together with the method of radioimmunoassay for oestradiol, have been previously described (Nicholson, *et al.*, 1978; Maynard & Nicholson, 1979). The protein content of cytosol fractions was estimated using Lowry's method.

### RESULTS

All tumours used in the study were classified according to their ER content. $ER^+$ tumours were defined as those tumours containing oestrogen-binding components in excess of 8 fmol/mg cytosol protein (Nicholson *et al.*, 1978). Using this classification scheme, 78% of tumours were $ER^+$. Data from these tumours are described in Figs. 1 and 2. The effects of giving twice-daily injections of either 5 or 0·5 μg of ICI 118630 resemble the effects of ovariectomy and tamoxifen treatment in that each was followed by a decrease in tumour volume (Fig. 1a, b, c and f). Reducing the dose of ICI 118630 to 0·05 μg decreased its capacity to promote substantial tumour regression.

Fig. 2 shows that after the cessation of ICI 118630 treatment in Group (a) (5 μg twice daily) no further growth was seen in 5 of the tumours (Fig. 2b). Of the remaining 10 tumours, 2 showed spectacular increases in tumour volume during the next 20-day period. Tumours which regrew after ICI 118630 treatment retained their hormone dependency and regressed after ovariectomy (Fig. 2c). Similar results were seen with tumours from Groups (b), (c) and (f) (not illustrated).

The data in Fig. 3*a* show the growth patterns of 7 $ER^-$ tumours during ICI 118630 (Groups (a) and (b) and tamoxifen treatment (Group (f)). Five of the tumours continued to grow during the treatment period and 2 regressed. In the 3 instances where ovariectomy occurred after the cessation of treatment, a progressive tumour growth pattern was maintained (Fig. 3b). During the study 2 fibroadenomas were detected, both being unresponsive to ICI 118630 treatment and ovariectomy (not illustrated).

In addition to the above effects of ICI 118630 on the growth of palpable tumours, the compound also decreased the

FIG. 2.—Growth patterns of tumours in animals after cessation of ICI 118630 treatment. (a) Animals bearing $ER^+$ DMBA-induced mammary tumours were administered 5 μg ICI 118630 twice daily for 20 days. Results are the mean ± range of values shown in Fig. 1a. (b) Tumour regrowth after the cessation of ICI 118630 treatment. (c) Secondary effects of ovariectomy on tumours growing after drug withdrawal.

FIG. 3.—Response of ER⁻ mammary tumours to treatment with ICI 118630 and tamoxifen. (a) Animals injected twice daily for 20 days with 5 μg ICI 118630 (●), or 0·5 μg ICI 118630 (○) or daily with 100 μg tamoxifen (■). (b) On cessation of treatment suitable animals were ovariectomized.

number of new tumours formed during treatment (Table I). The effect was obvious at each of the 3 dose levels. When treatment ceased, some growth of new tumours did occur, however, although the total number of tumours formed was less than in the control group. Fig. 4a details

TABLE II.—*Effect of* 118630 *and ovariectomy on plasma oestradiol*-17β *levels*

| Group | Dose/ injection (μg) | No. of animals | Mean plasma oestradiol levels (pg/ml) (and range) |
|---|---|---|---|
| (Control) | – | 6 | 33·6 (12·4–60·7) |
| (ICI 118630) | 5 | 6 | 8·6 (3·4–15·0) |
| (Ovariectomy) | – | 6 | 8·7 (3·5–24) |

the growth patterns of newly formed tumours after cessation of treatment in Groups (a) and (b). Of the 11 tumours examined, 9 were detected within 21 days of drug withdrawal. In the 7 where ovariectomy was possible, all newly developed tumours were hormone-dependent (Fig. 4b). Similar results were seen in the tamoxifen-treated group.

Twice-daily administration of 5 μg ICI 118630 for 20 days significantly reduced circulating oestradiol-17β levels in tumour-bearing animals (Table II). The results were indistinguishable from those in ovariectomized animals.

### DISCUSSION

The results described in this paper indicate that [D-Ser(Bu$^t$)$^6$Azgly$^{10}$]-LH-RH (ICI 118630) is a potent anti-tumour agent in DMBA-induced mammary tumours of the rat. Twice-daily administration of ICI 118630 (0·5 or 5 μg) is as effective as ovariectomy or tamoxifen (100 μg daily) in eliciting 2 phenomena: (1) decrease in the number of newly formed tumours; (2) regression of pre-existing tumours. In common with ovariectomy (McGuire *et al.*, 1971) and tamoxifen therapy (Jordan, 1975; Nicholson

TABLE I.—*Effect of ICI* 118630, *tamoxifen and ovariectomy on development of mammary tumours*

| Group | Treatment | Dose/injection (μg) | No. of animals | New tumours formed: During treatment | After treatment | Total |
|---|---|---|---|---|---|---|
| a | (ICI 118630) | 5·0 | 11 | 0 | 8 | 8 |
| b | (ICI 118630) | 0·5 | 7 | 0 | 3 | 3 |
| c | (ICI 118630) | 0·05 | 8 | 4 | 4 | 8 |
| d | (Control) | — | 7 | 16 | 2 | 18 |
| e | (Ovariectomy) | — | 9 | 2 | 1 | 3 |
| f | (Tamoxifen) | 100 | 9 | 3 | 4 | 7 |

FIG. 4.—Development of new tumours after ICI 118630 withdrawal. (a) Growth patterns of new tumours after the cessation of ICI 118630 (5·0 $\mu$g, ○; or 0·5 $\mu$g, ●) treatment. (b) Response to ovariectomy of new tumours.

*et al.*, 1978) the LH-RH agonist acts mainly on $ER^+$ tumours. $ER^-$ tumours showed an equivocal overall response to ICI 118630 treatment. This information, taken in conjunction with the observation that ICI 118630 decreases plasma oestradiol levels, suggests that the compound may act by eliciting chemical castration and thus depriving the tumour tissue of oestradiol.

Although attractive, the hypothesis does not include a role for prolactin, a hormone which has been implicated both individually and synergistically with oestrogen in the growth of DMBA-induced mammary tumours (Pearson *et al.*, 1972; Sinha *et al.*, 1973). Recently, Danguy and his colleagues (Danguy *et al.*, 1977) have suggested that the anti-tumour effects of another LH-RH agonist, [D-leu$^6$, des-gly $NH_2{}^{10}$, Pro-ethylamide$^9$]-LH-RH (A 43818) were mediated by reduction in the supply of prolactin to the target tissue. In their study, oestrogen secretion was apparently not suppressed, since oestrous cycles were still present.

In addition to the potent capacity of ICI 118630 (5 $\mu$g twice daily) to cause tumour regression of $ER^+$ rat mammary tumours, it is noteworthy that 5/16 tumours within the ICI 118630 treatment group showed no regrowth on withdrawal of the drug (Fig. 2b). Thus, within the strict limitations of the experiment, the LH-RH agonist "cured" several tumours. Where tumours did regrow, they were subsequently shown to be hormone-dependent. Hormone dependency was also characteristic of new tumours formed after ICI 118630 treatment had stopped. Such information suggests that the treatment cannot destroy all tumour cells. A pool of resting cells, immune to the lytic effects of the treatment, yet capable of proliferating and gaining hormone dependency in the absence of the drug, might explain this phenomenon. It will be of interest, therefore, to determine whether longer treatment might effect a higher "cure" rate by destroying tumour cells as they are processed through the resting phase into an active hormone-dependent state.

In conclusion we have noted that the LH-RH agonist ICI 118630 has extremely potent anti-tumour properties in the rat. The tumour responses produced by this compound are equal in magnitude to those after either ovariectomy or tamoxifen treatment. Since the latter two treatments are well established in the therapy of advanced breast cancer, it seems likely that ICI 118630 may also have therapeutic value.

We are grateful to ICI Ltd. Pharmaceutical Division for the opportunity to study the LH–RH analogue; to Mr B. G. Brownsey for hormone assays; to Professor K. Griffiths for his constructive criticism and advice; and to the Tenovus Organization and ICI Ltd. for financial support.

### REFERENCES

DANGUY, A., LEGROS, N., HEUSON-STIENNON, J. A., PASTEELS, J. I., ATASSI, G. & HEUSON, J. C. (1977) Effects of a gonadotrophin-releasing hormone (GnRH) analogue (A-43818) on 7,12-dimethylbenz(a)anthracene-induced rat mammary tumours. Histological and endocrine studies. *Eur. J. Cancer*, **18**, 1089.

DANIEL, P. M. & PRITCHARD, M. M. L. (1963) The response of experimentally induced mammary tumours in rats to hypophysectomy and pituitary stalk section. *Br. J. Cancer*, **17**, 446.

DUTTA, A. S., FURR, B. J. A., GILES, M. B., VALCACCIA, B. & WALPOLE, A. L. (1978*a*) Potent agonist and antagonist analogues of luliberin containing an azaglycine residue at Position 10. *Biochem. Biophys. Res. Comm.*, **81**, 382.

DUTTA, A. S., FURR, B. J. A., GILES, M. B. & VALCACCIA, B. (1978*b*) Synthesis and biological activity of highly active $\alpha$-aza-analogues of luliberin. *J. Med. Chem.*, **21**, 1018.

HUGGINS, C., BRIZIARELLI, G. & SUTTON, H. (1959) Rapid induction of mammary carcinoma in the rat and influence of hormones on the tumours, *J. Exp. Med.*, **109**, 25.

JORDAN, V. C. (1975) The anti-tumour effect of tamoxifen in the dimethylbenzanthracene-induced rat mammary carcinomata model. In *The Hormonal Control of Breast Cancer*, ICI Pharmaceuticals Publication. p. 11.

MAYNARD, P. V. & NICHOLSON, R. I. (1979) Biological effects of high dose levels of a series of new LH–RH analogues in intact female rats. *Br. J. Cancer*, **39**, 274.

MCGUIRE, W. L., JULIAN, J. & CHAMNESS, G. (1971) Dissociation betweeen ovary dependent growth and estrogen sensitivity in mammary carcinoma. *Endocrinology*, **89**, 969.

NICHOLSON, R. I., DAVIES, P. & GRIFFITHS, K. (1978) Tamoxifen binding in mammary tumours in relation to response. *Rev. Endocrine Related Cancer*. Supplement April, ICI Publications (UK) p. 306.

NICHOLSON, R. I. & GOLDER, M. P. (1975) The effect of synthetic antioestrogens on the growth and biochemistry of rat mammary tumours. *Eur. J. Cancer*, **11**, 571.

NICHOLSON, R. I., FINNEY, E. & MAYNARD, P. V. (1978) Activity of a new LH–RH analogue, ICI 118630, on the growth of rat mammary tumours. *J. Endocrinol.* **79**, 51.

PEARSON, O. H., MOLINA, A., BUTLER, T. P., LLERENA, L. & NASR, H. (1972) Estrogens and prolactin in mammary cancer. In *Estrogen Target Tissues and Neoplasia*, Ed T. L. Dao. Chicago: University Press. p. 287.

SINHA, D., COOPER, D. & DAO, T. L. (1973) Nature of estrogen and prolactin effect on mammary tumorigenesis. *Cancer Res.*, **33**, 411.


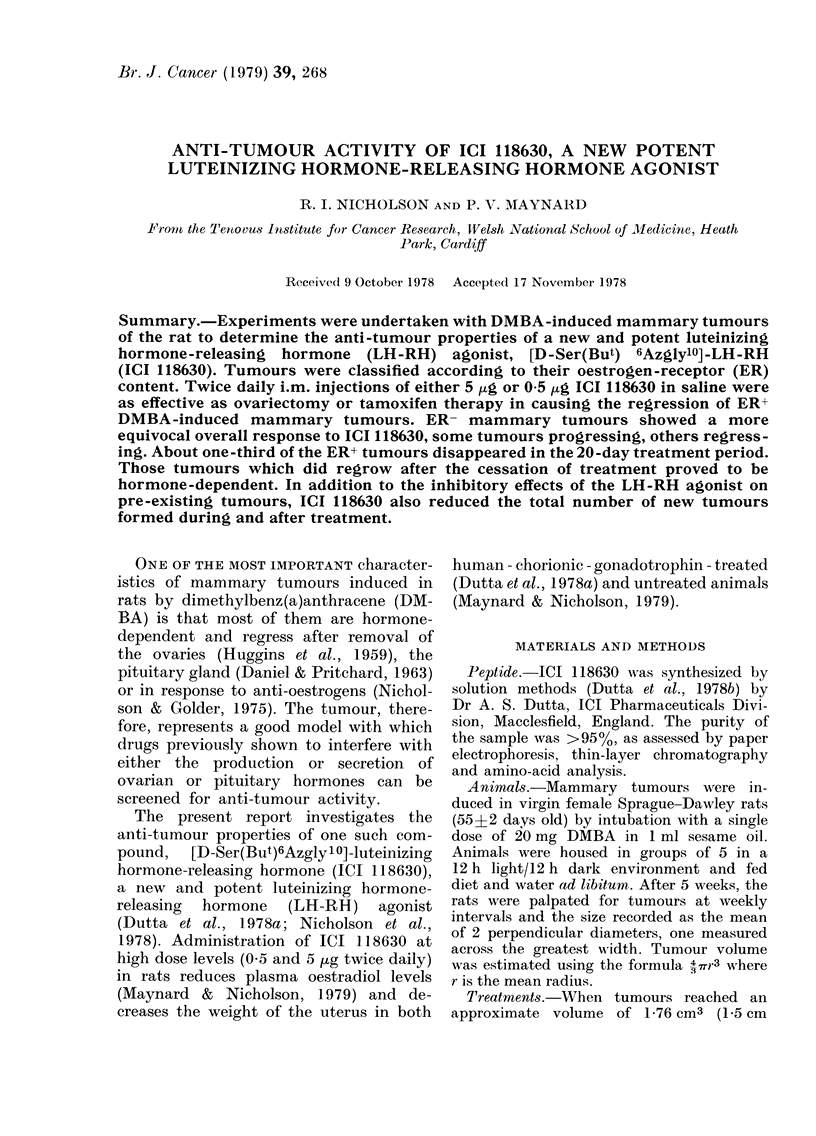

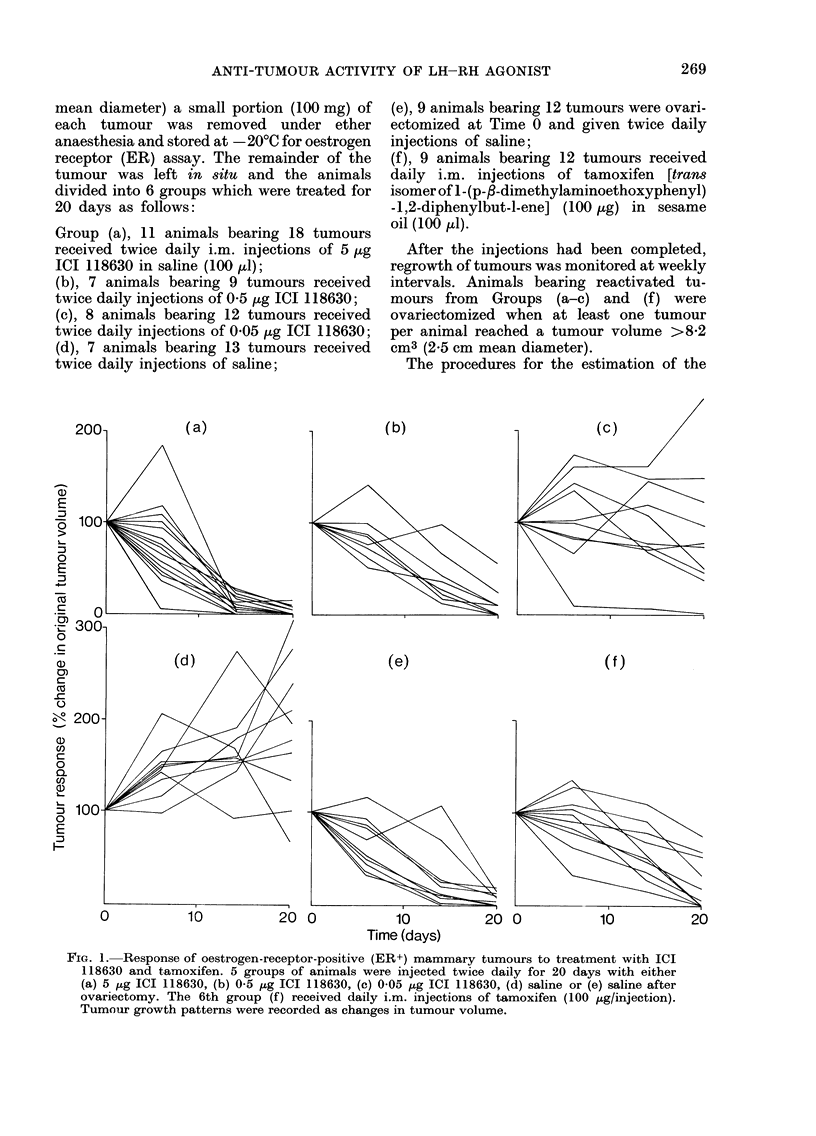

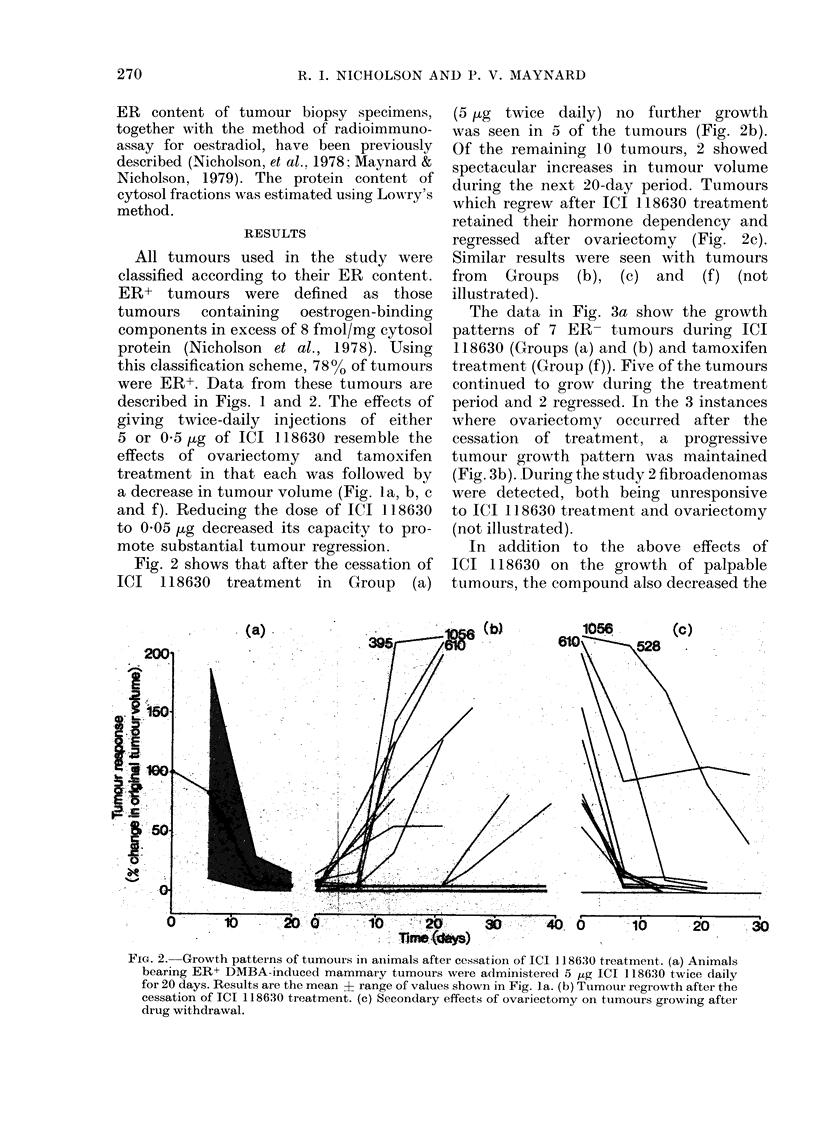

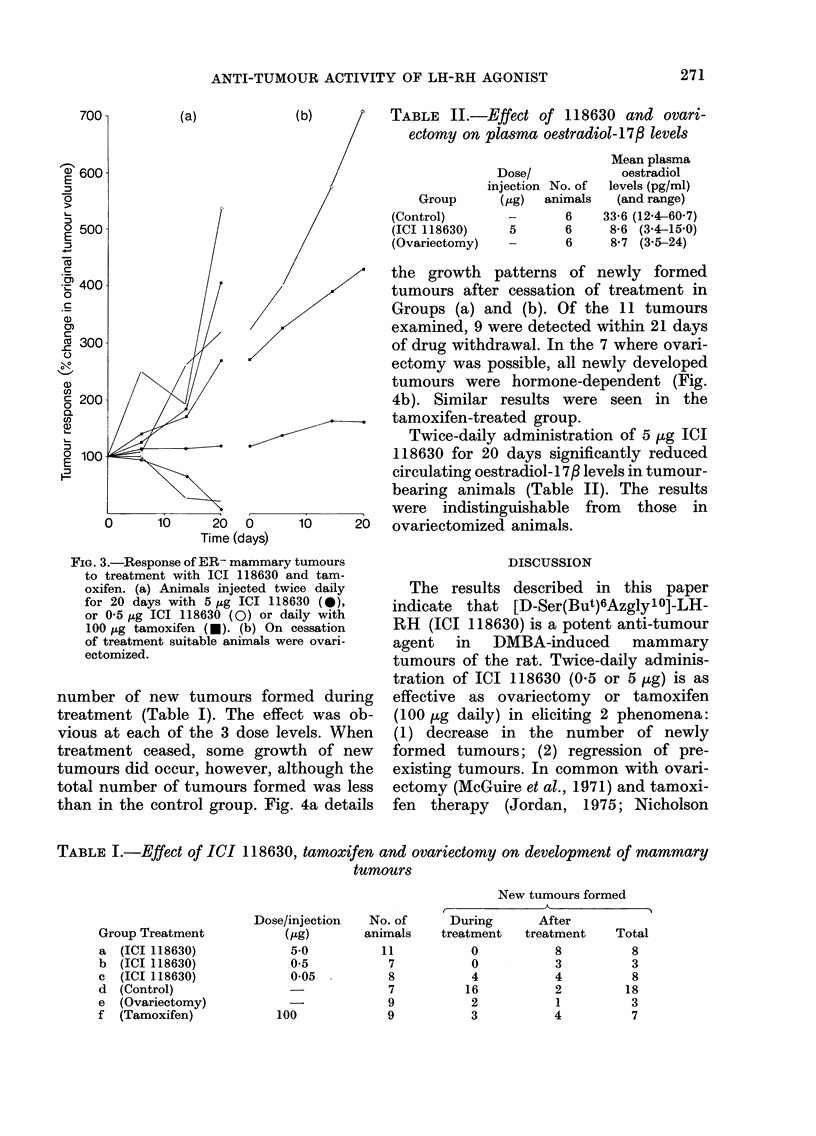

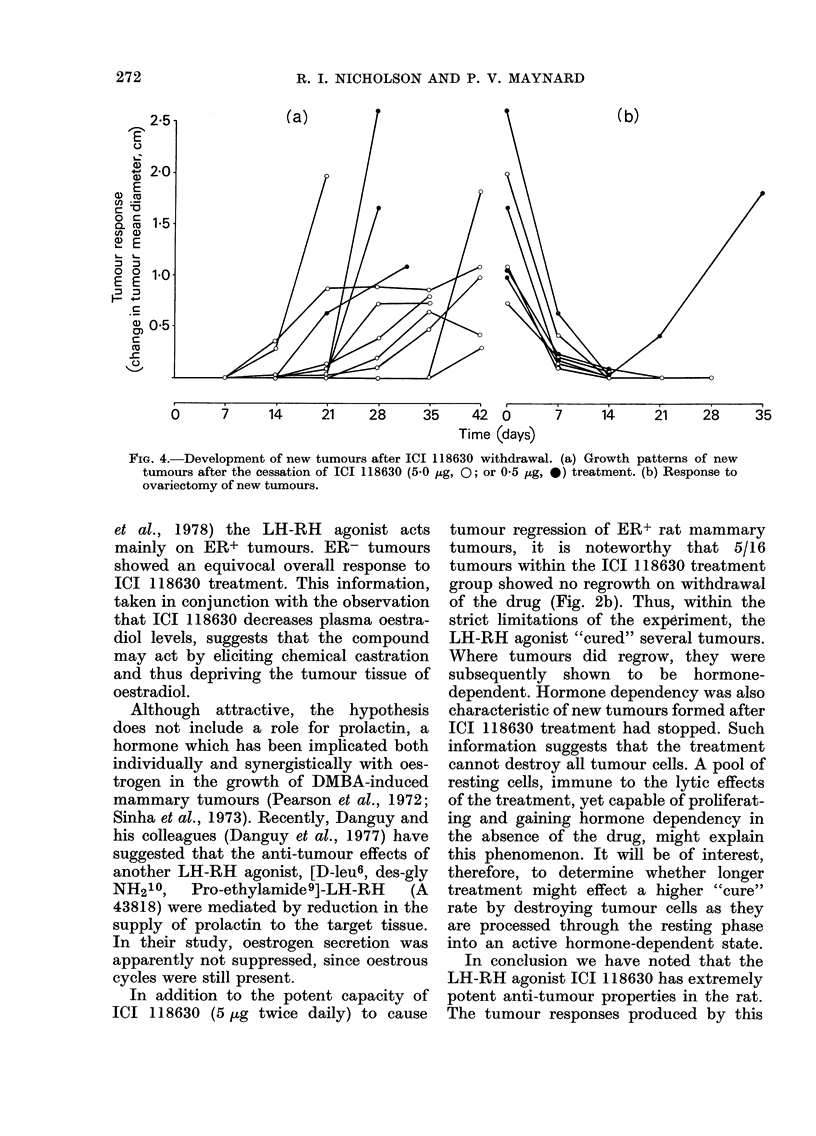

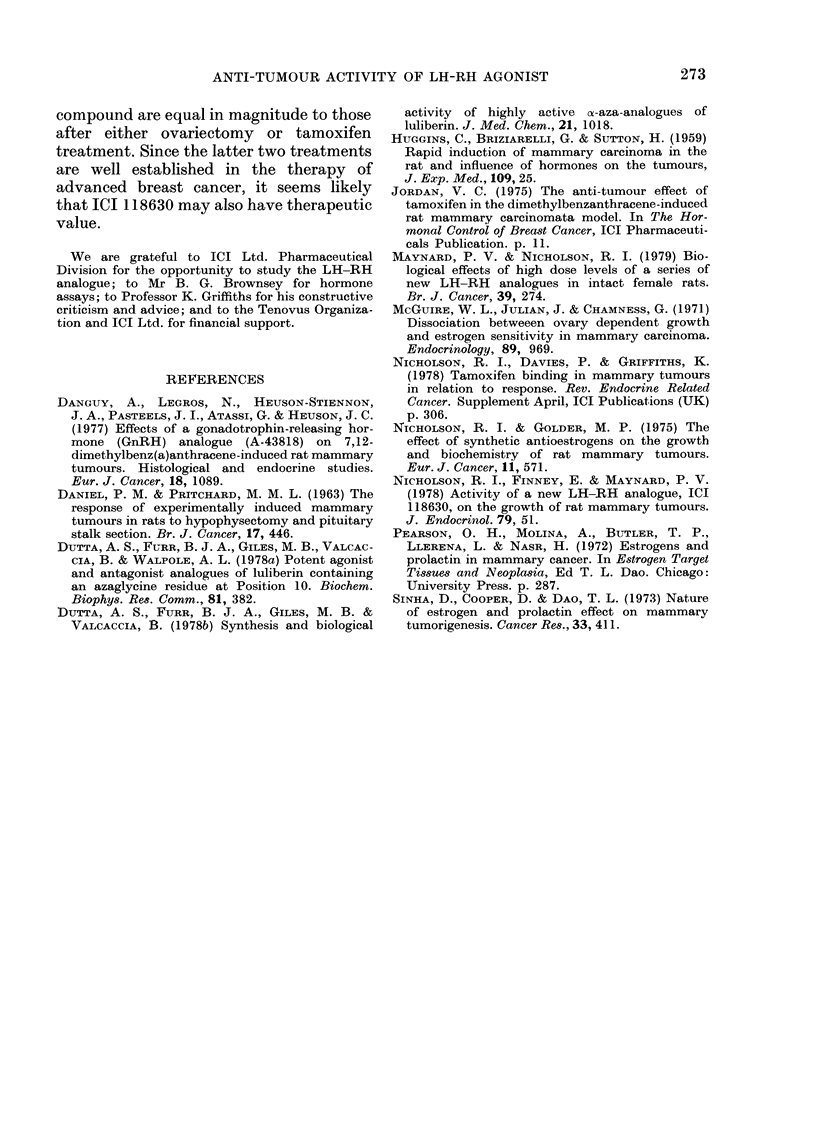


## References

[OCR_00603] DANIEL P. M., PRICHARD M. M. (1963). THE RESPONSE OF EXPERIMENTALLY INDUCED MAMMARY TUMOURS IN RATS TO HYPOPHYSECTOMY AND TO PITUITARY STALK SECTION.. Br J Cancer.

[OCR_00594] Danguy A., Legros N., Heuson-Stiennon J. A., Pasteels J. L., Atassi G., Heuson J. C. (1977). Effects of a gonadotropin-releasing hormone (GnRH) analogue (A-43818) on 7, 12- dimethylbenz (a)anthracene-induced rat mammary tumors. Histological and endocrine studies.. Eur J Cancer.

[OCR_00616] Dutta A. S., Furr B. J., Giles M. B., Valcaccia B. (1978). Synthesis and biological activity of highly active alpha-aza analogues of luliberin.. J Med Chem.

[OCR_00611] Dutta A. S., Furr B. J., Giles M. B., Valcaccia B., Walpole A. L. (1978). Potent agonist and antagonist analogues of luliberin containing an azaglycine residue in position 10.. Biochem Biophys Res Commun.

[OCR_00623] HUGGINS C., BRIZIARELLI G., SUTTON H. (1959). Rapid induction of mammary carcinoma in the rat and the influence of hormones on the tumors.. J Exp Med.

[OCR_00636] Maynard P. V., Nicholson R. I. (1979). Effects of high doses of a series of new luteinizing hormone-releasing hormone analogues in intact female rats.. Br J Cancer.

[OCR_00642] McGuire W. L., Julian J. A., Chamness G. C. (1971). A dissociation between ovarian dependent growth and estrogen sensitivity in mammary carcinoma.. Endocrinology.

[OCR_00655] Nicholson R. I., Golder M. P. (1975). The effect of synthetic anti-oestrogens on the growth and biochemistry of rat mammary tumours.. Eur J Cancer.

[OCR_00674] Sinha D., Cooper D., Dao T. L. (1973). The nature of estrogen and prolactin effect on mammary tumorigenesis.. Cancer Res.

